# Systemic Oxidative Stress and Visceral Adipose Tissue Mediators of NLRP3 Inflammasome and Autophagy Are Reduced in Obese Type 2 Diabetic Patients Treated with Metformin

**DOI:** 10.3390/antiox9090892

**Published:** 2020-09-21

**Authors:** Zaida Abad-Jiménez, Sandra López-Domènech, Rubén Díaz-Rúa, Francesca Iannantuoni, Segundo Ángel Gómez-Abril, Dolores Periañez-Gómez, Carlos Morillas, Víctor M. Víctor, Milagros Rocha

**Affiliations:** 1 Department of Endocrinology and Nutrition, University Hospital Doctor Peset, Foundation for the Promotion of Health and Biomedical Research in the Valencian Region (FISABIO), 46017 Valencia, Spain; zaiaji@alumni.uv.es (Z.A.-J.); Sandra.Lopez@uv.es (S.L.-D.); diaz_rub@gva.es (R.D.-R.); franian@alumni.uv.es (F.I.); carlos.morillas@uv.es (C.M.); 2 Department of General and Digestive System Surgery, University Hospital Doctor Peset, Foundation for the Promotion of Health and Biomedical Research in the Valencian Region (FISABIO), 46017 Valencia, Spain; gomez_seg@gva.es (S.Á.G.-A.); perianyez_dol@gva.es (D.P.-G.); 3 Department of Surgery, Faculty of Medicine and Dentistry, University of Valencia, Av Blasco Ibáñez 13, 46010 Valencia, Spain; 4 CIBERehd-Department of Pharmacology, University of Valencia, Av Blasco Ibáñez 13, 46010 Valencia, Spain; 5 Department of Physiology, Faculty of Medicine and Dentistry, University of Valencia, 46010 Valencia, Spain

**Keywords:** visceral adipose tissue (VAT), obesity, type 2 diabetes (T2D), inflammatory cytokines, autophagy, oxidative stress, metformin

## Abstract

Obesity is a low-grade inflammatory condition affecting a range of individuals, from metabolically healthy obese (MHO) subjects to type 2 diabetes (T2D) patients. Metformin has been shown to display anti-inflammatory properties, though the underlying molecular mechanisms are unclear. To study whether the effects of metformin are mediated by changes in the inflammasome complex and autophagy in visceral adipose tissue (VAT) of obese patients, a biopsy of VAT was obtained from a total of 68 obese patients undergoing gastric bypass surgery. The patients were clustered into two groups: MHO patients and T2D patients treated with metformin. Patients treated with metformin showed decreased levels of all analyzed serum pro-inflammatory markers (TNFα, IL6, IL1β and MCP1) and a downwards trend in IL18 levels associated with a lower production of oxidative stress markers in leukocytes (mitochondrial ROS and myeloperoxidase (MPO)). A reduction in protein levels of MCP1, NFκB, NLRP3, ASC, ATG5, Beclin1 and CHOP and an increase in p62 were also observed in the VAT of the diabetic group. This downregulation of both the NLRP3 inflammasome and autophagy in VAT may be associated with the improved inflammatory profile and leukocyte homeostasis seen in obese T2D patients treated with metformin with respect to MHO subjects and endorses the cardiometabolic protective effect of this drug.

## 1. Introduction

Obesity represents a risk factor for diverse clinical and metabolic disturbances, leading to increased mortality and shortened life expectancy. The predominant storage of fat in the visceral adipose tissue (VAT) depot characteristic of the abdominal obesity phenotype is typically accompanied by a wide range of metabolic abnormalities, including dyslipidemia, insulin resistance, hypertension and endothelial dysfunction [1,2]. These effects are exacerbated by the particularly aberrant release of cytokines and adipokynes by the VAT [3], which contributes to systemic low-grade inflammation, increased cardiovascular risk and development of type 2 diabetes (T2D).

The first-choice medical approach to achieve glycemic control and reduce cardiovascular risk factors in T2D patients is oral anti-diabetic medication. Metformin is the most commonly prescribed drug for T2D and is thought to exert its primary anti-diabetic action by suppressing hepatic glucose production and through a direct effect on cellular metabolism by inducing AMP-activated protein kinase (AMPK) and inhibiting mitochondrial reactive oxygen species (ROS) production through mitochondrial complex I. Metformin not only displays anti-hyperglycemic properties, but also improves endothelial function, lipid profile and hemostasis [4]. In addition, it is now attracting attention due to its role as an immune system modulator with anti-inflammatory properties [5,6,7], though the molecular mechanisms underlying these pleiotropic effects have not yet been determined. Emerging evidence suggests that metformin displays anti-inflammatory effects via direct and indirect targeting of tissue-resident immune cells in metabolic organs such as the liver, gastrointestinal tract and adipose tissue [8].

In contrast to obese T2D patients, who constitute a paradigm population of metabolic complications, there is a sub-cluster of “metabolically healthy” obese (MHO) subjects characterized by an absence of metabolic abnormalities. It is now thought that MHO is a transient phenotype that can turn into unhealthy metabolic obesity during the natural course of the disease [9]. Such patients are characterized by the storage of fat predominantly in the subcutaneous depot, less immune cell infiltration into the VAT depot, and a more favorable adipokine secretion pattern and inflammatory profile, including a lower release of tumor necrosis factor alpha (TNFα) and interleukin 1β (IL1β) [10,11,12] compared to that released by VAT. In this way, the inflammasome complex is a key component of our innate immune system, representing the backbone of the host defense and inflammatory response. Activation of the nucleotide-binding oligomerization domain (NOD), leucine-rich repeats (LRR) and pyrin domain-containing protein 3 (NLRP3) inflammasome usually requires both priming and an activation signal. The nuclear factor kB (NFκB) transcription factor, which is activated by Toll-like receptor (TLR) ligands and cytokines, such as TNFα and IL1β, is the main mediator of the priming signal, and acts by inducing the transcriptional expression of NLRP3 and pro-IL1β [13]. The second signal of inflammasome activation is triggered by various microbial components (pathogen-associated molecular patterns (PAMPs)) or molecules released by necrotic cells and damaged tissues (damage-associated molecular patterns (DAMPs)) [14], which are thought to activate NLRP3 by inducing various cellular events, including K^+^ efflux, Ca^2+^ signaling and mitochondrial and lysosomal damage, all of which release substances such as ROS, oxidized mitochondrial DNA and lysosomal proteases [15,16]. Metabolic abnormalities in obese patients are accompanied by higher levels of both immune cell infiltration and expression of NLRP3 and IL1β in VAT than those seen in MHO subjects [12,17]. However, whether metformin exerts its anti-inflammatory effect by modulating the activation of the inflammasome complex in the VAT of obese patients has been poorly studied to date [18,19].

Autophagy not only constitutes a mechanism of cell recycling necessary for cellular homeostasis and viability, but it is also increasingly recognized as an important component of both innate and acquired immunity to pathogens [20]. In this process, cytosolic macromolecules and damaged organelles are a target for autophagosomal capture following degradation by lysosome fusion [21]. This process involves a heterogeneous network of protein signaling pathways named mammalian autophagy-related (ATG) proteins, which are organized in six functional protein groups and proteins, including Beclin1, microtubule-associated protein 1A/1B-light chain 3 (LC3) and sequestosome 1 (p62) [22]. Since classic autophagy is known to be activated principally by pathways that mediate the nutrient deficiency/low energy state that induces AMPK and inhibits mechanistic target of rapamycin (mTOR) activity [20], it would be expected that obesity—a chronic state of nutrient overabundance—is associated with the downregulation of autophagy. However, there is strong evidence that autophagy levels are upregulated in obese adipocytes and adipose tissue explants [23,24,25], even in diabetic conditions, which leads to attenuated mTOR signaling [26]. Although metformin has been found to induce an upregulation of autophagy mediators in cardiomyocytes, melanoma cells and hepatocytes [27,28,29], few studies have investigated the effect of metformin on the induction of autophagy in adipose tissues [29,30].

Given the well-known contribution of VAT accumulation and the chronic inflammation and metabolic disturbances characteristic of diabetes and obesity, it is possible that the beneficial effects of metformin on the modulation of immune cell response, oxidative stress and subclinical low-grade inflammation [31,32] can be mediated by targeting adipose-specific activities. For this reason, the present study was undertaken to explore whether the underlying molecular mechanisms of the systemic anti-inflammatory and immune regulatory effects of metformin involve NLRP3 complex activation and/or modulation of the autophagy pathway in the VAT of obese subjects. Hence, we first aimed to confirm the effect of metformin on serum proinflammatory cytokines and systemic oxidative stress markers. Secondly, and more importantly, we evaluated the association between these systemic responses and VAT-specific pathways, including the NLRP3 inflammasome and autophagy.

## 2. Materials and Methods

### 2.1. Study Population

This was a transversal study of 68 obese patients between the ages 30 and 60 that were recruited from the Outpatient’s Department of Endocrinology and Nutrition at University Hospital Dr. Peset (Valencia, Spain). The patients were clustered into two groups: metabolically healthy obese (MHO) subjects and obese patients with T2D treated with metformin, all of whom underwent a laparoscopic Roux-en-Y gastric bypass (RYGB). The MHO group consisted of subjects who did not meet any of the following clinical criteria for metabolic syndrome—fasting glucose ≥ 100 mg/dL or use of anti-diabetic treatment, systolic blood pressure ≥ 130 mmHg and/or diastolic blood pressure ≥ 85 mmHg or use of antihypertensive drugs, triglyceride concentrations ≥ 150 mg/dL or high-density lipoprotein cholesterol (HDLc) < 40 mg/dL for men and < 50 mg/dL for women or the use of lipid-lowering medication [33] (*n* = 34). The T2D group was defined according to the criteria of the American Diabetes Association Guidelines [34] (*n* = 34). Exclusion criteria were severe disease (including malignancies, severe renal or hepatic disease, alcohol or drug abuse and psychiatric disorders), history of cardiovascular or chronic inflammatory disease, and secondary obesity (hypothyroidism and Cushing’s syndrome).

The study protocol was approved by the Ethics Committee of the hospital (reference code 96/16) and was regulated according to the guidelines set out in the Declaration of Helsinki. Written informed consent was obtained from all subjects.

### 2.2. Clinical and Biochemical Determinations

Anthropometric measures—weight, height, waist circumference, body mass index (BMI) and systolic (SBP) and diastolic blood pressure (DBP)—were obtained by physical examination. Blood samples were collected from the antecubital vein in fasting conditions between 8:00 a.m. and 9:30 a.m. for biochemical and molecular determinations. To obtain serum, centrifugation was performed at 650× *g* for 10 min at 4 °C. Glucose, total cholesterol (TC) and triglyceride (TG) serum levels were determined by enzymatic assay (Beckman Corp. Brea, CA, USA). The percentage of HbA1c was obtained with an automatic glycohemoglobin analyzer (Arkray inc., Kyoto, Japan). HDLc concentration was measured using a Beckman LX20 analyzer (Brea, CA, USA) and low-density lipoprotein cholesterol (LDLc) levels were calculated with Friedewald’s formula. Insulin levels were measured by immunochemiluminescence (Abbott, Chicago, IL, USA) and the homeostatic model assessment of the insulin resistance (HOMA-IR) index was calculated as ((fasting insulin (µU/mL) × fasting glucose (mg/dL))/405). High-sensitivity C-reactive protein (hsCRP) was analyzed using a latex-enhanced immunonephelometric assay (Behring Nephelometer II; Dade Behring, Inc., Newark, DE, USA) and leukocytes were counted with a COULTER^®^ LH 500 Hematology Blood Analyzer from Beckman Coulter (Brea, CA, USA). All biochemical determinations were performed in the hospital’s Clinical Analysis Department.

### 2.3. Cell Isolation

Citrated blood samples were incubated with dextran (3%) for 45 min in order to isolate polymorphonuclear leukocytes (PMNs). The supernatant, layered over Ficoll-Hypaque (GE Healthcare, Uppsala, Sweden), was then centrifuged at 650× *g* for 25 min. Lysis buffer was added to remove the erythrocytes remaining in the pellet. PMNs were washed twice and re-suspended in Hanks’s balanced salt solution (HBSS; Sigma Aldrich, MO, USA). Finally, PMNs were counted with a Scepter 2.0 cell counter (Millipore, MA, USA).

### 2.4. Analysis of Oxidative Stress Markers and Serum Cytokines

To detect mitochondrial ROS, leukocytes were seeded in a 48-well plate (1.5 × 10^5^ cells/well) and incubated for 30 min with MitoSOX dye (Life Technologies, Thermo Fisher Scientific, Waltham, MA, USA) prepared at 5 μM in HBSS. The nuclei were visualized using the nuclear stain Hoechst 33342. The fluorescence signal was detected with an IX81 Olympus fluorescence microscope and analyzed with ScanR software version 2.03.2 (Olympus, Shinjuku, Japan).

Serum levels of interleukin 6 (IL6), IL1β, interleukin 18 (IL18), TNFα and myeloperoxidase (MPO) were measured with a Luminex^®^ 200 analyzer system (Austin, TX, USA) following the Milliplex^®^ MAP Kit manufacturer’s procedure (Millipore Corporation, Billerica, MA, USA). The intra-serial and inter-serial variation coefficients were <5.0% and <20.0%, respectively.

### 2.5. VAT Protein Analysis

During RYGB surgery, approximately 1.5–2.0 g VAT biopsies were retrieved from the omentum area and immediately frozen in liquid nitrogen and stored at −80 °C. For protein isolation, 150 mg of VAT were homogenized with Ultra-Turrax^®^ in the protein lysis buffer provided by the Ne-Per^®^ Kit (Thermo Fisher Scientific, Waltham, MA, USA) in the presence of protease and phosphatase inhibitors (Sigma Aldrich, St. Louis, MO, USA). Following the manufacturer’s protocol, samples were centrifuged twice at 15,000× *g* for 20 min at 4 °C to remove superficial fat. The total concentration of proteins was quantified using a bicinchoninic acid (BCA) protein assay (Thermo Fisher Scientific, Waltham, MA, USA).

To determine protein expression levels, 25 µg of protein samples were resolved on polyacrylamide gels and then transferred onto nitrocellulose membranes. Next, membranes were blocked and incubated overnight at 4 °C with the following primary antibodies: monoclonal anti-NFκB (R&D Systems, Minneapolis, MN, USA), monoclonal anti-monocyte chemoattractant protein 1 (MCP1; Santa Cruz Biotechnology, Santa Cruz, CA, USA), monoclonal anti-NLRP3 (Cell Signaling Technology, Danvers, MA, USA), monoclonal anti-apoptosis-associated speck-like protein containing a C-terminal caspase recruitment domain (CARD) (ASC; Santa Cruz Biotechnology, Santa Cruz, CA, USA), monoclonal anti-autophagy related protein 5 (ATG5; Cell Signaling Technology, Danvers, MA, USA), monoclonal anti-Beclin1 (Abcam, Cambridge, UK), monoclonal anti-p62 (Santa Cruz Biotechnology, Santa Cruz, CA, USA), monoclonal anti-CCAAT enhancer-binding protein homologous protein (CHOP; Thermo Fisher Scientific, Waltham, MA, USA). Monoclonal anti-actin (Sigma-Aldrich, St. Louis, MO, USA) was used to assess loading protein control. Horseradish peroxidase (HRP) -goat anti-mouse (Thermo Fisher Scientific, Waltham, MA, USA) and HRP-goat anti-rabbit (Millipore Iberica, Madrid, Spain) were employed as secondary antibodies. Membranes were exposed to ECL Plus reagent (GE Healthcare, Little Chalfont, UK) or SuperSignal West Femto (Thermo Fisher Scientific, Waltham, MA, USA). The chemiluminescence signal was detected with the Fusion FX5 acquisition system and quantified by densitometry using Bio1D software version 15.03a (Vilbert Lourmat, Marne La Valleé, France).

### 2.6. Statistical Analysis

SPSS 20.0 software (IBM SPSS Statistic, Chicago, IL, USA) was used for data analysis. The parametric data shown in the tables are expressed as mean ± SD, whereas non-parametric data are shown as median and interquartile range (25th and 75th percentiles). Bar graphs show mean + SEM. Data were compared with an unpaired Student’s *t*-test or Mann–Whitney U test for parametric and non-parametric variables, respectively. Pearson’s correlation coefficient was employed to determine the degree of relationship between the study variables. Possible confounding variables, such as age, were used as covariates to generate a univariate general linear model for analyzing biochemical parameters and serum lipids. A confidence interval of 95% was used for all the tests, and differences were considered significant when *p* < 0.05.

## 3. Results

### 3.1. Anthropometric and Biochemical Parameters

Our study analyzed anthropometric and biochemical parameters and prescribed medication in 68 obese patients, most of whom were women, and all of whom underwent RYGB surgery (Table 1). All the diabetic patients were taking metformin as an oral anti-diabetic drug; 62% were under hypotensive medication, 67% were on lipid-lowering drugs and 35% were receiving all three drugs.

The diabetic group treated with metformin had higher waist circumference (*p* < 0.01), waist-to-hip ratio (WHR) (*p* < 0.01), SBP (*p* < 0.001), DBP (*p* < 0.05) and TG (*p* < 0.01) than MHO subjects, whereas BMI distribution, leukocyte count, hsCRP and lipid profile were similar in the two groups (Table 1). As expected, T2D patients treated with metformin displayed significant differences in glucose metabolism parameters, including elevated levels of glucose (*p* < 0.001), insulin (*p* < 0.05), HOMA-IR (*p* < 0.01) and HbA1c (*p* < 0.001) with respect to the MHO group. Finally, although diabetic patients were generally older, differences remained significant when we introduced age as a covariant in the general linear model (Table 1).

### 3.2. Systemic Oxidative Stress Markers and Pro-Inflammatory Cytokines

**Figure 1:**
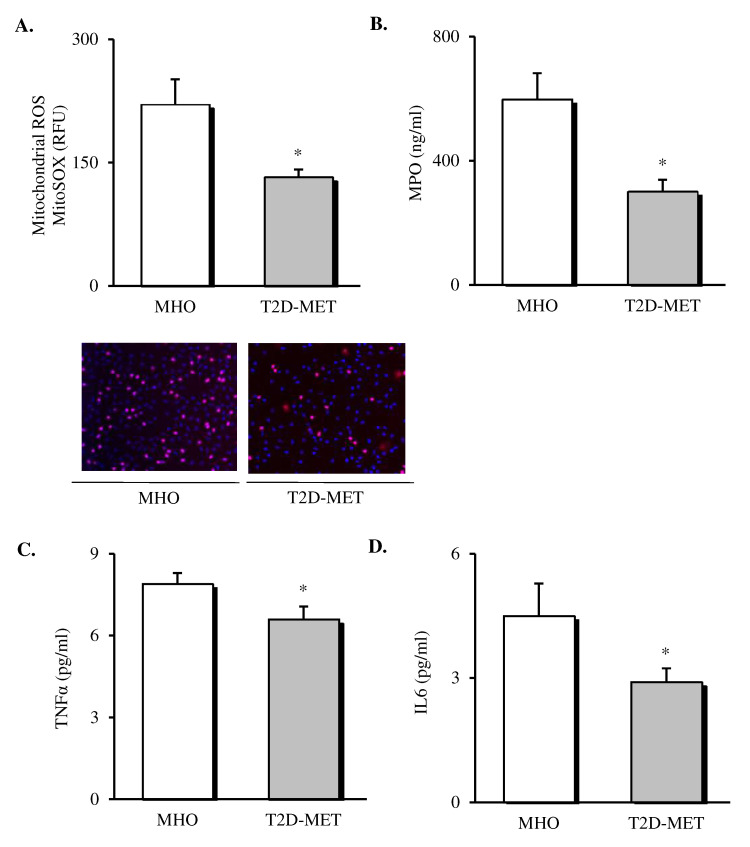
Evaluation of systemic oxidative stress markers and cytokines in the serum of MHO and obese T2D patients treated with metformin. Levels of mitochondrial ROS production (**A**) expressed as arbitrary units and the representative images of their leukocytes stained with MitoSox (red) and Hoechst 33,342 (blue) and visualized by fluorescence microscopy (*n* = 20 in MHO and *n* = 17 in T2D-Met), serum levels of (**B**) MPO (*n* = 11 in MHO and *n* = 16 in T2D-Met), (**C**) TNFα (*n* = 14 in MHO and *n* = 15 in T2D-Met) and (**D**) IL6 (*n* = 12 in MHO and *n* = 15 in T2D-Met). Data are expressed as mean + standard error. * *p* < 0.05 when compared using unpaired Student’s *t*-test. ROS, reactive oxygen species; RFU, relative fluorescence units; MPO, myeloperoxidase; IL6, interleukin 6; TNFα, tumor necrosis factor alpha; MHO, metabolically healthy obese; T2D-Met, type 2 diabetic patients treated with metformin.

Oxidative stress markers and cytokines were assessed in the leukocytes and serum of patients (Figure 1). The results showed a decrease in the production of mitochondrial ROS (Figure 1A, *p* < 0.05) in leukocytes of diabetic patients treated with metformin. In addition, we determined MPO levels in serum, since it is a potent pro-oxidant, derived mainly from neutrophils, that mediates vascular damage. The results showed that MPO was markedly reduced in the diabetic population (Figure 1B, *p* < 0.05), although their total leukocyte count remained unchanged (Table 1), thus suggesting a reduced MPO release by the leukocyte defense system. Moreover, systemic levels of TNFα and IL6 were also lower in T2D patients treated with metformin (Figure 1C,D, *p* < 0.05).

### 3.3. NLRP3 Inflammasome Complex Activation in VAT

**Figure 2:**
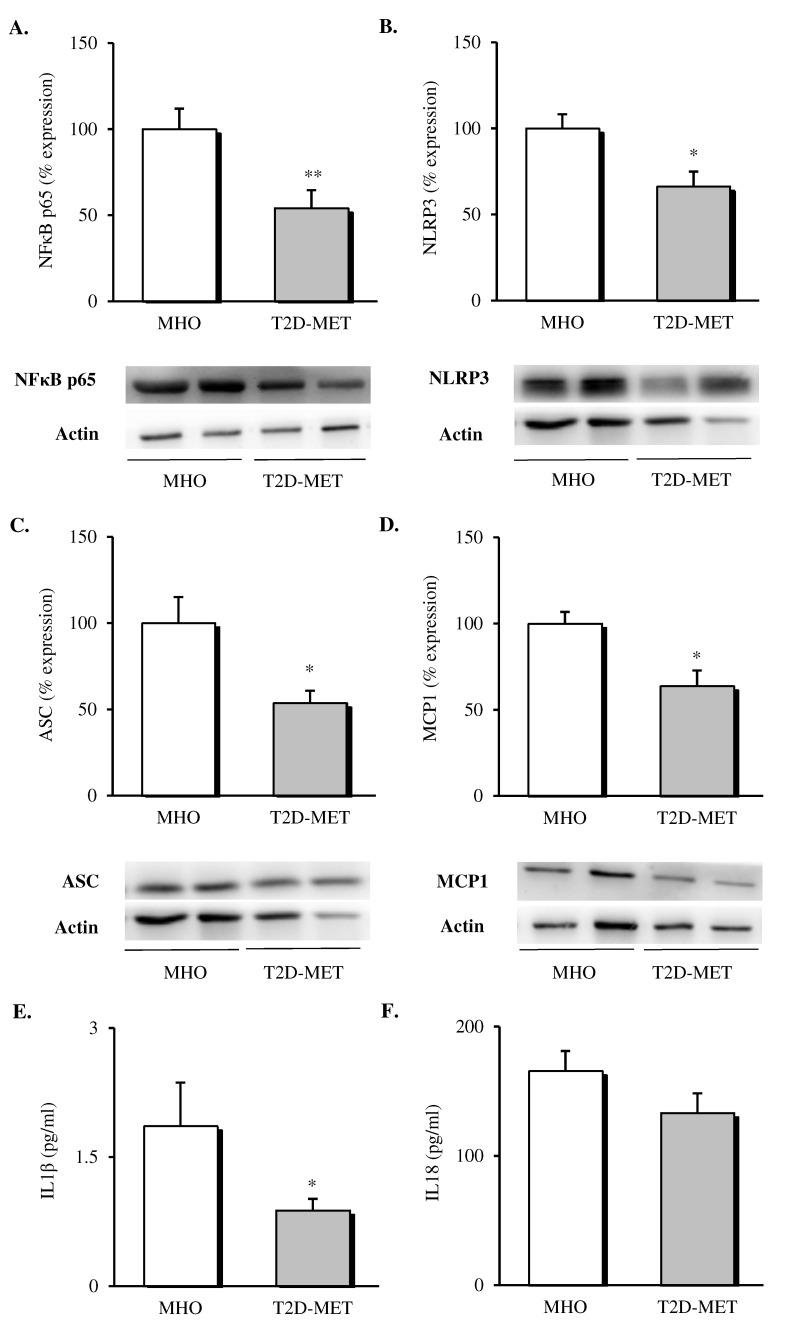
Evaluation of inflammasome complex mediators and interleukins in the VAT and serum of MHO and obese T2D patients treated with metformin. Relative protein expression and representative western blot images of the inflammatory transcription factor (**A**) NFκB (*n* = 9 in MHO and *n* = 10 in T2D-Met), inflammasome complex components (**B**) NLRP3 (*n* = 11 in MHO and *n* = 9 in T2D-Met) and (**C**) ASC (*n* = 12 per group), (**D**) MCP1 (*n* = 9 in MHO and *n* = 13 in T2D-Met) and serum levels of (**E**) IL1β (*n* = 10 in MHO and *n* = 14 in T2D-Met) and (**F**) IL18 (*n* = 13 per group). Data are expressed as mean + standard error. * *p* < 0.05 and ** *p* < 0.01 when compared using unpaired Student’s *t*-test. VAT, visceral adipose tissue; MHO, metabolically healthy obese; T2D-Met, type 2 diabetic patients treated with metformin; NFκB, nuclear factor kB; MCP1, monocyte chemoattractant protein 1; NLRP3, NACHT, LRR and PYD domain-containing protein 3; ASC, apoptosis-associated speck-like protein containing a caspase recruitment domain; IL1β, interleukin 1 beta; IL18, interleukin 18.

The inflammatory status of VAT was evaluated by assessing the expression of different proteins of the NLRP3 inflammasome complex and pro-inflammatory mediators. Firstly, we observed a decrease in the protein expression levels of all the analyzed markers in the VAT of diabetic patients treated with metformin (Figure 2). Specifically, our results showed a decline in the percentage of the expression of NFκB (Figure 2A, *p* < 0.01), NLRP3 (Figure 2B, *p* < 0.05) and ASC (Figure 2C, *p* < 0.05) in the metformin-treated group compared to MHO subjects, which was accompanied by a significant drop in the expression of the chemoattractant signal MCP1 (Figure 2D, *p* < 0.05).

When we evaluated serum levels of cytokines released by the formation of the NLRP3 inflammasome complex, a significant decrease in serum IL1β (Figure 2E, *p* < 0.05) and a downward trend in serum IL18 (Figure 2F, *p* = 0.146) were observed in diabetic patients treated with metformin with respect to MHO subjects.

### 3.4. Protein Expression of Autophagy Mediators in VAT

**Figure 3:**
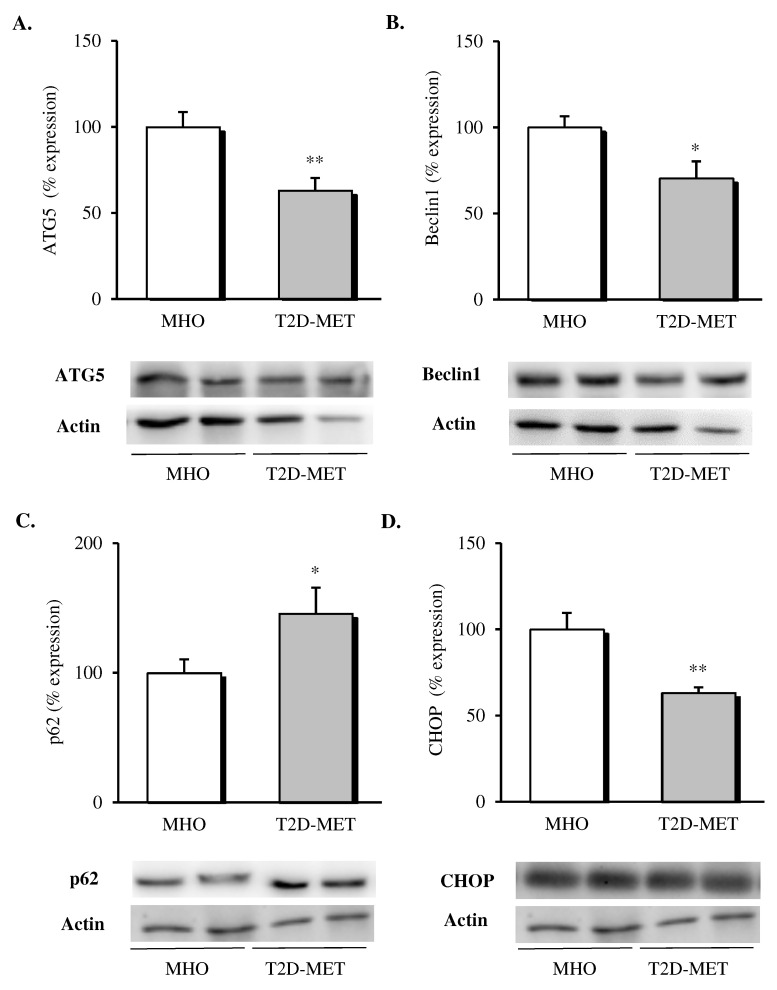
Evaluation of autophagy mediators and CHOP in the VAT of MHO and obese T2D patients treated with metformin. Relative protein expression and representative western blot images of (**A**) ATG5 (*n* = 12 in MHO and *n* = 13 in T2D-Met), (**B**) Beclin1 (*n* = 12 in MHO and *n* = 13 in T2D-Met), (**C**) p62 (*n* = 12 in MHO and *n* = 11 in T2D-Met) and proapoptotic marker (**D**) CHOP (*n* = 12 in MHO and *n* = 10 in T2D-Met). Data are expressed as mean + standard error. * *p* < 0.05 and ** *p* < 0.01 when compared using unpaired Student’s *t*-test. CHOP, CCAAT/enhancer-binding protein (C/EBP) homologous protein; VAT, visceral adipose tissue; MHO, metabolically healthy obese; T2D-Met, type 2 diabetic patients treated with metformin; ATG5, autophagy related 5.

When different autophagy markers were analyzed in VAT, a general downregulation of this pathway was observed in patients treated with metformin (Figure 3); in particular, lower protein levels of ATG5 (Figure 3A, *p* < 0.01) and Beclin1 (Figure 3B, *p* < 0.05) and significantly higher p62 levels (Figure 3C, *p* < 0.05) were detected when compared with MHO subjects. Finally, the proapoptotic marker CHOP was also evaluated, showing a significant drop (Figure 3D, *p* < 0.01) in the diabetic group, in line with the improved adipose tissue functionality.

### 3.5. Analysis of Correlations between Variables

Pearson’s correlation studies revealed a significant and positive correlation between mitochondrial ROS in leukocytes and systemic MPO (*r* = 0.665, *p* = 0.004, *n* = 17). When we analyzed the inflammatory profile, systemic cytokine IL1β was positively associated with VAT expression of ATG5 (*r* = 0.598, *p* = 0.031, *n* = 13). Both IL1β and IL6 were negatively correlated with the autophagic flux protein p62 (*r* = −0.577, *p* = 0.049, *n* = 12; *r* = −0.682, *p* = 0.010, *n* = 13), and the expression of the inflammatory mediator NFκB in VAT was positively associated with Beclin1 (*r* = 0.613, *p* = 0.005, *n* = 19), ATG5 (*r* = 0.664, *p* = 0.003, *n* = 18), NLRP3 (*r* = 0.626, *p* = 0.017, *n* = 14) and ASC (*r* = 0.813, *p* = 0.000, *n* = 18). At the same time, NLRP3 and ASC both correlated with the autophagic markers ATG5 (*r* = 0.809, *p* = 0.000, *n* = 19; *r* = 0.734, *p* = 0.000, *n* = 23, respectively) and Beclin 1 (*r* = 0.614, *p* = 0.005, *n* = 19; *r* = 0.574, *p* = 0.004, *n* = 23, respectively). Additionally, MCP1 expression was positively associated with ATG5, Beclin1 and NLRP3 (*r* = 0.631, *p* = 0.002, *n* = 21; *r* = 0.542, *p* = 0.011, *n* = 21 and *r* = 0.606, *p* = 0.008, *n* = 18, respectively). When we evaluated the expression of the proapoptotic protein CHOP, we observed a positive correlation with ATG5 (*r* = 0.535, *p* = 0.012, *n* = 21), Beclin1 (*r* = 0.458, *p* = 0.032, *n* = 22) and mediators of the inflammasome complex NFκB (*r* = 0.537, *p* = 0.026, *n* = 17), NLRP3 (*r* = 0.655, *p* = 0.003, *n* = 18) and serum IL1β (*r* = 0.605, *p* = 0.037, *n* = 12).

## 4. Discussion

In the present study, we demonstrate that obese T2D patients treated with metformin display a more favorable immuno-inflammatory status than MHO subjects: namely, a reduction in leukocyte ROS production, an improved systemic inflammatory profile, and an amelioration in the activation of the inflammasome complex, autophagy and apoptosis mediators measured in VAT. In the context of its emerging role as an immune system modulator, we suspect that metformin mediates these anti-inflammatory responses and improves the cardiometabolic profile of T2D patients, even with respect to obese individuals that do not apparently exhibit overt metabolic abnormalities.

Consistent data show that mitochondria are the main subcellular target of metformin, wherein it reduces ROS production as a direct consequence of mitochondrial complex I suppression [4]. In line with this, our present data reveal a decrease in mitochondrial ROS production by leukocytes of obese diabetic patients treated with metformin compared with MHO subjects. These results are in apparent conflict with our previous results in which T2D patients showed higher ROS production than MHO subjects [35]. However, there are differences between the two study designs that need to be taken into consideration. In the study by Bañuls et al., previous metformin treatment was not considered as an inclusion criterion, and patients with T2D displayed higher BMI than those with MHO, a characteristic we have previously shown to increase oxidative stress [36]. In addition, metformin treatment reduced mitochondrial ROS and increased antioxidant levels with respect to non-metformin-treated T2D patients [37], which is in accordance with our present findings and those of Bułdak et al., who showed that macrophages treated with metformin express less ROS and display increased antioxidant activity and reduced inflammatory cytokine production [38].

Simultaneously, MPO, which is stored mainly in neutrophils, can also contribute to vascular injury and oxidative stress, thus exacerbating the inflammatory response [39]. Our data support these findings, as they show a positive correlation between MPO and mitochondrial ROS production in leukocytes. Among the few studies published about the effect of metformin on MPO, one reported that metformin significantly reduced MPO activity in pulmonary tissue [40], which is in accordance with our findings.

Under conditions of obesity, adipose tissue and leukocytes are crucial mediators of a chronic low-grade inflammatory state, since they secrete proinflammatory mediators and contribute to insulin resistance [41]. Previous clinical studies have shown that metformin reduces systemic inflammatory markers such as IL6 and TNFα in obese and T2D patients [42,43]. Furthermore, it has been demonstrated that metformin diminishes TNFα and MCP1 production in adipose tissue in a rodent obesity model with insulin resistance [18]. MCP1 is an adipocytokine that is crucial for triggering macrophage infiltration into adipose tissue and recruiting more macrophages to sites of infiltration, where these activated macrophages further stimulate MCP1 production. In a model of hypertrophied adipocytes, MCP1 protein was shown to be significantly inhibited by treatment with metformin by a mechanism involving the NFκB pathway [44]. As a whole, these previous results are in line with our present findings, and together they confirm a role for metformin in ameliorating systemic inflammation, with a specific action in adipose tissue, where it reduces expression of MCP1 and NFκB.

As mentioned previously, NFκB is the main mediator of the priming signal of the NLRP3 inflammasome. Thus, the inhibition of NFκB expression could be involved in downregulation of the NLRP3 inflammasome [45]. In fact, when compared with obese subjects without metabolic complications, our obese diabetic patients treated with metformin exhibited a significantly lower expression of the inflammasome components NRLP3 and ASC and a drop in the systemic release of IL1β and IL18, the specific cytokines resulting from NLRP3 inflammasome activation. This response could have been mediated by the well-known effect of metformin on NFκB and ROS decline [44,46]. In line with this, Li et al. reported that metformin prevented NLRP3 inflammasome activation by suppressing endoplasmic reticulum (ER) stress, indicated by dephosphorylation of IRE1α and eIF2α in the adipose tissue of diabetic mice [19]. These findings are consistent with the reduced expression of the proapoptotic factor CHOP observed in the present study, which is a downstream signal of chronic ER stress activation. Furthermore, we have previously reported that leukocytes from patients treated with metformin present impaired production of NLRP3 and reduced levels of IL1β and IL18 compared with non-diabetic subjects [47], which is also in line with our present findings.

Several studies have reported a crosstalk between autophagy and inflammasome activation in macrophages, suggesting that the former accompanies the latter as an autoregulatory mechanism to restrain its pro-inflammatory function [48,49]. Accordingly, we observed that NLRP3 and ASC were both positively correlated with ATG5 and Beclin1, while systemic IL1β showed a positive association with ATG5 and a negative association with p62, which is constantly degraded during the autophagic process [50]. This endorses a positive correlation between activation of the inflammasome complex and autophagy.

Compelling evidence suggest that metformin could display its effect on the regulatory mechanism of autophagy in a tissue-dependent fashion. Thus, in a diet-induced obesity model, metformin was shown to improve autophagy in the liver, while it was inhibited in the epididymal adipose tissue [29], which is in line with the inhibition of autophagy in VAT observed in the present work, and mediated by a significant drop in protein expression of Beclin 1 and ATG5 and an increase in p62 (autophagy initiation, elongation and cargo recognition, respectively). In contrast, autophagy has been shown to increase progressively in epididymal adipose tissue in a lean mouse model after metformin treatment [30]. We can speculate about whether these differential responses to metformin treatment could be modulated by obesity. Indeed, autophagy is regulated in a defective manner in the hypertrophic adipose tissue of mice and humans. In a study in lean mice, caloric deprivation was shown to result in a significant increase in the expression of markers of autophagy in VAT, while it was reduced in obese mice. A similar response has been found in human obese subjects, in which body mass reduction led to an attenuation of autophagy in subcutaneous adipose tissue [51].

One of the main strengths of the present study is that we included an MHO group without any cardiometabolic risk or pharmacological treatment. Moreover, as far as we know, this is the first study to report a modulation of the NLRP3 inflammasome and autophagy activation in the VAT of obese patients treated with metformin. Indeed, the significant correlation between these specific tissue markers and circulating cytokines IL6 and IL1β supports the modulation of these molecular pathways in highly metabolically active VAT as contributors to the systemic anti-inflammatory effect of metformin.

Although these results undoubtedly broaden our understanding of the global action of metformin, the transversal nature of the study limits the inference of causality, and therefore more mechanistic studies are required to confirm this notion. One weakness of the study is the lack of a group of drug-naive diabetic patients, though it should be stressed that patients undergoing RYGB are closely monitored with respect to medication. In addition, we chose to carry out our analyses in VAT, which is composed of a diverse cell population that includes adipocytes, vascular stromal cells and various immune cells with an important role in obesity. In this way, whole-tissue changes may mask specific regulation of molecular pathways in a particular cell population and/or may reflect changes in the cellular composition of the tissue.

## 5. Conclusions

To summarize, our findings provide evidence that obese T2D patients treated with metformin exhibit an improved inflammatory and oxidative status with respect to MHO subjects. This improvement seems to be modulated by changes in the activation of the inflammasome complex and autophagy in VAT, suggesting—despite common belief—that MHO subjects are not as cardiometabolically protected as expected. Future mechanistic studies should aim to determine the direct targets of metformin responsible for mediating these responses.

## Figures and Tables

**Table 1:** Anthropometric and biochemical parameters of the study cohort.

Parameters	MHO	T2D-Met	Corrected by Age
*n* (females %)	34 (85.3)	34 (70.6)	
Age (years)	38.1 ± 9.1	51.8 ± 9.1 ***	
BMI (kg/m^2^)	38.8 ± 4.3	39.6 ± 4.6	*p* > 0.05
Waist (cm)	111 ± 11	120 ± 12 **	*p* < 0.01
Waist-to-hip ratio	0.86 ± 0.06	0.93 ± 0.10 **	*p* < 0.05
SBP (mmHg)	120 ± 9	138 ± 20 ***	*p* < 0.001
DBP (mmHg)	76 ± 7	84 ± 15 *	*p* < 0.05
Glucose (mg/dL)	90 ± 8	119 ± 37 ***	*p* < 0.001
Insulin (μU/mL)	12.90 ± 6.11	18.18 ± 9.76 *	*p* < 0.001
HOMA-IR	2.96 ± 1.60	5.81 ± 9.12 **	*p* < 0.001
HbA1c (%)	5.24 ± 0.32	6.20 ± 0.88 ***	*p* < 0.001
TC (mg/dL)	182 ± 37	181 ± 42	*p* > 0.05
HDLc (mg/dL)	46 ± 8	45 ± 9	*p* > 0.05
LDLc (mg/dL)	116 ± 31	107 ± 36	*p* > 0.05
TG (mg/dL)	85 (66, 109)	122 (98, 163) **	*p* < 0.05
hsCRP (mg/L)	4.79 (3.42, 11.05)	4.00 (1.34, 7.98)	*p* > 0.05
Leukocytes (cells × 10^3^/μL)	7.3 ± 1.8	7.8 ± 1.9	*p* > 0.05
Treatment *n* (%)			
Hypertension	-	21 (62%)	
Hyperlipidemia	-	23 (67%)	
T2D	-	34 (100%)	

Data are expressed as mean ± SD or n (percentage). TG and hsCRP are represented in median and interquartile ranges (25% and 75% percentile). Values were statistically compared with an unpaired Student’s *t*-test or Wilcoxon’s test, and were considered significantly different when * *p* < 0.05 ** *p* < 0.01 and *** *p* < 0.001. BMI, body mass index; SBP, systolic blood pressure; DBP, diastolic blood pressure; HbA1c, glycated hemoglobin; TC, total cholesterol; LDLc, Low-density lipoprotein cholesterol; HDLc, high-density lipoprotein cholesterol; TG, triglycerides; hsCRP, high-sensitivity C-reactive protein; MHO, metabolically healthy obese; T2D-Met, type 2 diabetic patients treated with metformin.
